# The Influence of *FTO, FABP2, LEP, LEPR,* and *MC4R* Genes on Obesity Parameters in Physically Active Caucasian Men

**DOI:** 10.3390/ijerph19106030

**Published:** 2022-05-16

**Authors:** Ewelina Maculewicz, Agata Leońska-Duniec, Andrzej Mastalerz, Ewa Szarska, Aleksandra Garbacz, Tomasz Lepionka, Roman Łakomy, Anna Anyżewska, Jerzy Bertrandt

**Affiliations:** 1 Faculty of Physical Education, Jozef Pilsudski University of Physical Education in Warsaw, 00-809 Warsaw, Poland; andrzej.mastalerz@awf.edu.pl; 2 Faculty of Physical Education, Gdansk University of Physical Education and Sport, 80-336 Gdansk, Poland; agata.leonska-duniec@awf.gda.pl; 3 Military Institute of Hygiene and Epidemiology, 01-163 Warsaw, Poland; eszarska@gmail.com (E.S.); tomasz.lepionka@wihe.pl (T.L.); roman.lakomy@interia.pl (R.Ł.); 4 Institute of Animal Sciences, Faculty of Animal Breeding, Bioengineering and Conservation, Warsaw University of Life Sciences—SGGW, 02-787 Warsaw, Poland; aleksandra_garbacz@sggw.edu.pl; 5 University of Economics and Human Sciences in Warsaw, Okopowa 59, 01-043 Warsaw, Poland; anna.anyzewska@gmail.com; 6 Faculty of Health Sciences, Pope John Paul II State School of Higher Education in Biala Podlaska, 21-500 Biala Podlaska, Poland; jwbertrandt@gmail.com

**Keywords:** genes, SNPs, obesity-related traits, obesity risk, physical activity, Caucasian men

## Abstract

Obesity is a complex multifactorial abnormality that has a well-confirmed genetic basis. However, the problem still lies in identifying the polymorphisms linked to body mass and composition. Therefore, this study aimed to analyze associations between *FTO* (rs9939609), *FABP2* (rs1799883), and *LEP* (rs2167270), *LEPR* (rs1137101), and *MC4R* (rs17782313) polymorphisms and obesity-related parameters. Unrelated Caucasian males (*n* = 165) were recruited. All participants had similar physical activity levels. The participants were divided into two groups depending on their body mass index (BMI) and fat mass index (FMI). All samples were genotyped using real-time polymerase chain reaction (real-time PCR). When tested individually, only one statistically significant result was found. The *FTO* A/T polymorphism was significantly associated with FMI (*p* = 0.01). The chance of having increased FMI was >2-fold higher for the *FTO* A allele carriers (*p* < 0.01). Gene–gene interaction analyses showed the additional influence of all investigated genes on BMI and FMI. In summary, it was demonstrated that harboring the *FTO* A allele might be a risk factor for elevated fat mass. Additionally, this study confirmed that all five polymorphisms are involved in the development of common obesity in the studied population and the genetic risk of obesity is linked to the accumulation of numerous variants.

## 1. Introduction

Obesity is defined as excessive body weight gain due to an increased accumulation of body fat that presents a leading cause of the largest public health problems [1], since nearly 40% of adults are overweight and 10–15% are obese worldwide [2]. Obesity phenotypes are associated with a higher risk of many medical problems such as cardiovascular events, metabolic disorders, type 2 diabetes, neuropsychiatric disorders, and some types of cancer, the majority of which can lead to elevated mortality risk [3,4,5,6]. An analysis of the latest work in the literature has shown that modifiable factors positively affect our well-being and health. The best effect can be achieved by combining positive actions in the field of diet, supplementation, moderate but systematic physical activity, and adding the right amount of sleep per day [7,8].

Obesity is a complex multifactorial disease that has a well-confirmed strong genetic basis but needs behavioral, developmental, and/or environmental influences to develop [1,9,10]. Several studies have demonstrated the role of lifestyle, including caloric intake and physical activity level, in the regulation of body weight [11,12]. However, the problem still lies in identifying the genes and polymorphic sites related to obesity and describing the biological mechanisms by which they exert their effects [9].

Although studies of the common obesity genetics were determined by genome-wide association studies (GWASs), this stage was set by research on monogenic obesity, which emphasized that the leptin–melanocortin signaling pathway is the major regulator of food intake. Several genes involved in the development of monogenic obesity are in or near loci subsequently linked by GWASs with obesity-related traits. To date, over 600 genes and chromosomal regions have been associated with the regulation of body weight and composition [9,13]. The genetic risk of common obesity is linked to the accumulation of numerous loci, each contributing a small part of the total obesity risk [9]. Therefore, the analysis of haplotypes and interactions between candidate genes are more informative than methods based on individual signal-nucleotide polymorphism (SNP) and might give additional information important for understanding complex interactions between various gene variants [14,15]. Consequently, in our study, we decided to analyze five of the most promising polymorphic sites localized within obesity-related genes: fat mass and obesity-associated (*FTO*), fatty acid-binding protein 2 (*FABP2*), leptin (*LEP*), leptin receptor (*LEPR*), and melanocortin-4 receptor (*MC4R*), which are characterized in Table 1 [9,16,17,18,19,20,21,22]. The genes were selected based on a literature review and our own previous studies. Recently, studies have confirmed that these SNPs are linked to obesity-related traits such as BMI, hip circumference, total body weight, body fat percentage, and cardiometabolic traits, among others. The noted associations are replicable across different ethnic populations as well as various age groups [9,13,16,17,18,19,20,21,22]. Guilherme et al. (2019) suggested that SNPs, which can affect body composition parameters, might influence physical performance [23].

The above-mentioned findings highlight the selected genes’ association with body mass and body composition parameters. However, the results are inconsistent and the interactions between these candidate genes are still unknown. Thus, this study aimed to examine the associations between *FTO* (rs9939609), *FABP2* (rs1799883), *LEP* (rs2167270), *LEPR* (rs1137101), and *MC4R* (rs17782313) polymorphisms and obesity-related traits. Therefore, we studied individually and in gene–gene interaction models the alleles and genotypes distribution in a group of physically active Caucasian men measured for selected body mass and body composition traits.

## 2. Materials and Methods

### 2.1. Participants 

One hundred and sixty-five unrelated military professionals were selected for this study. All participants were aged 19–54 years, ancestrally fitted (all volunteers were Polish and Eastern Europe residents for 3 generations), and they represented similar physical activity levels. The experimental protocols used in this study were conducted in accordance with the World Medical Association’s Declaration of Helsinki and were positively verified by the Ethics Committee of the Military Institute of Hygiene and Epidemiology (no. 1/XXI /2016). The participants received an information sheet regarding the research details, aim of the study and procedures applied, as well as potential risks and benefits associated with their participation. All volunteers gave written, informed consent for the genotyping, and they were informed that the study will be anonymous and the results would be private.

### 2.2. Body Composition Measurements

Height was measured using a portable stadiometer (without shoes) (TANITA HR-001, Tanita Corporation, Tokyo, Japan). Body composition (including fat %) and body weight were measured using bioelectrical impedance analysis (BIA) using the TANITA MC-780 machine (Tanita Corporation, Japan) with an accuracy of 0.1 kg according to the procedure specified in the instruction manual (lightly dressed, without shoes). All measurements were performed according to the procedure specified in the instruction manual and without any metal objects. The following parameters were noted: BMI (kg/m^2^), height (cm), weight (kg), fat mass index (FMI; kg/m^2^) [24], visceral tissue index (VTI; level), and fat percentage (%). 

The participants (*n* = 165) were divided into two groups depending on their BMI (body weight/height^2^; kg/m^2^). The control group (CON_BMI_; *n* = 77) comprised people with BMI between 20.0 and 25.0, while the overweight group (OVER_BMI_; *n* = 88) had a BMI of ≥25.0. They were also divided into two groups depending on their FMI (fat mass/height^2^; kg/m^2^). FMI values between 3 and 6 were classified as normal fat mass; FMI lower than 3 —fat deficit; FMI higher than 6—excess fat. Participants whose FMI values were 6 and lower were classified into the CON_FMI_ group (*n* = 124), while those whose FMI values were higher than 6 were grouped into the OVER_FMI_ group (*n* = 41). Statistically significant differences between participants in both groups were observed for parameters BMI (kg/m^2^), age (years), weight (kg), FMI (kg/m^2^), VTI (level) and Fat(%) (*p*-value < 0.01). No statistically significant difference was shown in the parameter height (cm) (*p*-value = 0.81, 0.34) (Table 2). Detailed characteristics of experimental groups are given in Table 2.

### 2.3. Genetic Analyses

The buccal cells of the participants were collected using swabs (Copan FLOQSwabs, Interpath, Murrieta, Australia). Genomic DNA was extracted from the donated buccal cells using a High Pure PCR Template Preparation Kit (Roche Diagnostics, Munich, Germany) according to the manufacturer’s protocols. DNA samples of good quality and quantity were stored at -20 °C. The exclusion criteria were: failure in DNA extraction; DNA degradation; abnormal gene detection results; incomplete basic information. All samples were genotyped in duplicate, using TaqMan Pre-De-signed SNP Genotyping Assays, which are given in Table 3 (Applied Biosystems, Waltham, MA, USA) on a CFX Connect Real-Time PCR Detection System (Bio-Rad, Hercules, CA, USA) according to the manufacturer’s recommendations. The assays contained primers and fluorescently labeled (FAM and VIC) minor groove binder (MGB) probes. The real-time PCR conditions were as follows: 5 min of initial denaturation (95 °C), then 40 cycles of denaturation (15 s, 95 °C) and annealing/extension (60 s, 60 °C).

### 2.4. Statistical Analyses

All statistical analyses were performed using the program R (version 2.0-1, The R Foundation for Statistical Computing; https://cran.r-project.org (accessed on 20 September 2021)). Anthropometric data are shown as mean values ± standard deviation and differences among experimental groups were analyzed with Student’s t-test which was statistically significant when *p* < 0.05. To check the compliance of the variables with the normal distribution, the Shapiro–Wilk test was used and Levene’s test was used for verification of the homogeneity of variance. Single-locus analysis was performed considering four genetic models (codominant, dominant, recessive and overdominant) and was calculated with the SNPassoc package for R. The models were constructed concerning the minor allele and were checked adjusted by age as a potential factor influencing the result. FDR-adjusted *p*-values were calculated with the fdrtool package for R. An odds ratio (OR) was used as a measure of association between an exposure and an outcome and to determine whether a particular genotype is a risk factor for being overweight. The Akaike information criterion (AIC) was used to evaluate how well a model fits the data. A genetic model-free Multifactor dimensionality reduction (MDR) was used to detect the influence of the common effect of gene × gene interactions on BMI and FMI, and it was calculated with MDR3.0.2 (http://sourceforge.net/projects/mdr/ (accessed on 20 September 2021)); chi-square test was used for checking the statistical significance of the model. Balance accuracy was used as the evaluation measure to rank potential models and cross-validation consistency was used to choose the best models. The association of single alleles with BMI was calculated with Pearson’s chi-squared test with the STAT package for R. The level of statistical significance was set at the level of *p* < 0.05. Genotype frequencies were analyzed using Fisher’s exact test with the STAT package for R.

## 3. Results

All genotype frequencies did not significantly different from the Hardy–Weinberg equilibrium expectations in the OVER_BMI_ group (*p*-values range from 0.10 to 1.00), CON_BMI_ group (*p*-values range from 0.50 to 1.00), OVER_FMI_ (*p*-values range from 0.56 to 1.00), CON_FMI_ (*p*-values range from 0.47 to 1.00), and the case–control group (*p*-values range from 0.33 to 1.00; Table 4).

No significant association was found between *FTO* (rs9939609), *FABP2* (rs1799883), *LEP* (rs2167270), *LEPR* (rs1137101), *MC4R* (rs17782313) and the BMI value exceeding 25. All divisions were checked under four genetic models: codominant, dominant, recessive, and overdominant, *p*-values were between 0.11 and 0.98. The influence of single alleles on BMI division was also checked and no significant association was found (Table 5).

The *FTO* gene polymorphism (rs9939609) was significantly associated with FMI exceeding 6 (Table 6). An association was found for the codominant (AA vs. TT), dominant (AT-AA vs. TT), and for the recessive genetic models (AA vs. TT-AT). The chance of being OVER_FMI_ for the combination AA was over 4.7 times greater than for the combination TT in the codominant model (Fisher’s exact test *p* = 0.01). The chance of being OVER_FMI_ for the combination AT-AA was >2.7 times higher than for the combination TT in the dominant model (Fisher’s exact test *p* = 0.02). The chance of being OVER_FMI_ for the combination AA was >2.8 times higher than for the combination TT-AT in the codominant model (Fisher’s exact test *p* = 0.02). Moreover, the chance of being OVER_FMI_ was >2.0-fold higher for A allele with Pearson’s chi-squared test *p*-value < 0.01. The model was supplemented with age as a potential factor influencing the result, because of statistical differences shown between groups for this variable (Table 6).

Gene–gene interactions’ influence on BMI and FMI division was calculated with the MDR function. The best two-locus model in all divisions was that involving *FTO* (rs9939609) and *LEPR* (rs1137101), indicating a potential gene–gene interaction between these two genes. For BMI division, when genotypes AT × AA, TT × AA, TT × AG (*FTO* × *LEPR*, respectively) appear the model sorts the observations to join the CON_BMI_ group with a higher probability than joining the OVER_BMI_ group (*p* = 0.02). For FMI division when the genotypes AT × GG, TT × AA, and TT × AG (*FTO* × *LEPR*, respectively) appear, the model sorts the observations to join the CON_FMI_ group with a higher probability than joining the OVER_FMI_ group (*p* < 0.01; Table 7).

The best chosen three-locus model for BMI division involved the genes *LEP* (rs2167270), *LEPR* (rs1137101), and *MC4R* (rs17782313). The genotypes GG × AA × TT, GG × AG × TC, GG × AG × CC, GG × GG × TT, AA × AA × TT, AA × AA × TC, AA × AG × TT, AA × AG × CC, AG × AA × TT, AG × AG × TT, and AG × GG × CC (*LEP* × *LEPR* × *MC4R*, respectively) were selected by an algorithm to join the CON_BMI_ group (*p* < 0.01). For FMI division, the best three-locus model included the genes *FTO* (rs9939609), *LEPR* (rs1137101), and *MC4R* (rs17782313). The genotypes AT × GG × AA, AT × AG × GG, TT × GG × AG, TT × AG × AA, TT × AG × AG, and TT × AA × AG (*FTO* × *LEPR* × *MC4R*, respectively) were selected by an algorithm to join the control group with a higher probability (*p* < 0.01). When the genotypes TT × GG × GG, and TT × AA × AA (*FTO* × *LEPR* × *MC4R*, respectively) appeared, the model sorted the observations to join the CON_FMI_ group (*p* < 0.01). In all divisions in the four-locus and five-locus models when genotype was chosen to join the control group in *FTO* (rs9939609), only TT and AT genotypes appeared (Table 7).

## 4. Discussion

In the present study, we examined the allele and genotype distributions of the *FTO* (rs9939609), *FABP2* (rs1799883), *LEP* (rs2167270), *LEPR* (rs1137101), and *MC4R* (rs17782313) polymorphisms in a group of Caucasian men, who were divided into groups depending on their BMI and FMI. When tested individually, our statistical analyses showed that harboring the specific *FTO* genotype might be associated with FMI, which measures relative fat content. This association was found for the codominant (A/A vs. T/T), dominant (A/T-A/A vs. T/T), and also for the recessive genetic model (A/A vs. T/T-A/T). The chance of having an increased FMI was over two times higher for the *FTO* A allele carriers. This observation constitutes the first important finding of the present study, implying that harboring this specific allele is unfavorable for some individuals. The carriers of the A allele might have an increased accumulation of body fat and a higher risk of many obesity-related disorders. 

In 2007, three independent groups demonstrated that a cluster of polymorphisms in the *FTO* first intron was strongly related to body mass and composition parameters and predisposes to overweight and obesity in children, teenagers, and adults [25,26,27]. Since then, many studies have proved that the *FTO* variants, especially the common A/T polymorphism (rs9939609), are significantly linked to obesity-related traits, e.g., BMI, FMI, body fat percentage, hip circumference, cardiometabolic traits, and many obesity-related medical problems. These associations are found across various ethnic populations and different age groups [18,25,26,27,28,29]. The A allele, identified as the risk allele, is linked to increased appetite and reduced satiety, a higher intake of dietary protein and fat, poor eating habits, and loss of control overeating, among others [18]. Consequently, it has been linked to the development of overweight and obesity, increasing the risk by 20–30%. About 16% of examined individuals are homozygous for the A alleles and these people weigh ~3 kg more than those without the risk allele [24]. In a previous study including 201 young women from Poland, the A allele was also associated with higher BMI [29]. Additionally, Zmijewski and Leońska-Duniec showed that the SNP within the *FTO* gene can influence athlete status in a study involving 196 elite swimmers and 379 control participants, who were all Caucasians. They found that harboring the T allele might be favorable for achieving success in sports such as swimming [30]. These results are in accordance with our study, which confirms that harboring the A allele is unfavorable for Polish men, who might have an increased accumulation of body fat. These results suggest that the *FTO* (rs9939609) polymorphism is a candidate marker for affecting body mass and body composition parameters in the Caucasian population.

A few potential biological mechanisms underlying the relationship between the *FTO* polymorphism and body mass and composition parameters have been revealed. The research has indicated that these associations are mediated through their functional interactions with distal surrounding genes. The first intron of the *FTO* gene contains a binding site for the transcriptional factor—cut-like homeobox 1 (CUX1). Through controlling retinitis pigmentosa GTPase regulator interacting protein 1 (RPGRIP1L) expression, it interacts with the leptin receptor [27,31]. The leptin signaling is mediated by this specific receptor, which in turn regulates food intake and energy expenditure [16]. Additionally, the *FTO* intron also includes an enhancer sequence, which interacts with the iroquois homeobox 3 (IRX3) promoter region, and thus the *FTO* SNPs regulate IRX3 expression in the human brain. The IRX3 relationship with obesity and the process of browning in adipose cells has been described [27,31,32]. Currently, some studies have indicated that the *FTO* gene is closely related to the regulation of levels of growth hormone and insulin-like growth factor I (IGF-1). IGF-1 is a crucial hormone in the development of metabolic syndrome, due to its influence on lipid and carbohydrate metabolism [33].

When the results obtained in our study were incorporated into the complex gene–gene interaction analysis, the novel finding was that all five studied polymorphisms are involved in the formation of obesity-related traits in the Caucasian population. These results imply that some individuals might benefit from carrying some combinations of genotypes. It was shown that for the two-locus model—*FTO* × *LEPR* interaction—both the BMI and FMI division genotypes, TT × AA and TT × AG, were associated with the absence of overweight. The same result showed the genotypes AT × AA with BMI division and AT × GG for FMI division. For three-locus model genotypes, GG × AA × TT, GG × AG × TC, GG × AG × CC, GG × GG × TT, AA × AA × TT, AA × AA × TC, AA × AG × TT, AA × AG × CC, AG × AA × TT, AG × AG × TT, and AG × GG × CC (*LEP* × *LEPR* × *MC4R*) showed a link to the lack of overweight in BMI division. The same association was shown for genotypes AT × GG × AA, AT × AG × GG, TT × GG × AG, TT × AG × AA, TT × AG × AG, and TT × AA × AG (*FTO* × *LEPR* × *MC4R*) in FMI division. For four-locus and five-locus models, only the genotypes TT and AT in *FTO* present when there was an association with the absence of overweight shown (both for BMI and FMI). In all models, when *FTO* was included, the genotypes TT and AT were linked with a lack of overweight, confirming that harboring the *FTO* T allele might be favorable for some individuals.

Although the analysis of individual SNPs showed only one association between harboring the specific *FTO* genotype and FMI, the gene–gene interaction analysis revealed numerous links between the genotypes of studied genes and obesity-related traits. This observation confirmed that the genetic risk of obesity is connected with the accumulation of numerous variants; thus, methods based on numerous SNPs are more informative than methods based on a single polymorphism. Cole et al. showed that the analysis of gene–gene interactions is a potential source of unexplained heritability, a significant focus of research into complex traits, including obesity, which involves a complex interaction between several genetic variants. Such polygenic traits frequently require etiologies in which complicated biological relations within different tissues, pathways, and networks underlie the trait development [15]. Studying gene–gene interactions has been especially important in the context of obesity [34], which is in accordance with our results.

Although numerous studies refer to the analysis of individual SNPs, which were selected for the present study, the literature on gene–gene interaction analyses is scarce. Therefore, the obtained results cannot be discussed with direct comparisons to other studies. In the study including 2386 individuals, De et al. analyzed interactions between twelve genetic variants robustly associated with obesity (*BDNF*, *ETV5*, *FAIM2*, *FTO*, *GNPDA2*, *KCTD15*, *MC4R*, *MTCH2*, *NEGR1*, *SEC16B*, *SH2B1*, and *TMEM18*). The authors underlined that the used methodology made it possible to reveal the background of interactions between genes known to influence BMI. They characterized the complicated interactions, emphasized new roles of the genes and highlight the involvement of regulatory frameworks in the development of obesity; e.g., rs17066891 in *MC4R* was identified as having the strongest main effect within this network, rs9940128 in *FTO* was identified as having the second strongest main effect in the network, and rs4358154 in *TMEM18* had the highest score for all measures which highlights the potentially significant role of this variant in the context of obesity [34]. In a study including 290 overweight/obese participants and 288 normal-weight controls, Rana et al. examined the effects of gene–gene and gene–environment interactions on the obesity risk in the Pakistani population. They analyzed the five obesity-associated genetic variants (*MC4R* rs17782313, *BDNF* rs6265, *FTO* rs1421085, *TMEM18* rs7561317, and *NEGR1* rs2815752). Surprisingly, the gene–gene interaction was not found to significantly influence any obesity-related anthropometric phenotype, such as BMI or body fat percentage [35].

The study group was very homogeneous, which is a strong point of our study. Participants had the same living conditions, physical activity levels, and meals, and yet the differences in the parameters related to body weight were statistically significant. This allows most of the environmental factors to be ruled out and might indicate a genetic background for the increased body weight. A potential limitation of our experiment is rather the small group size of the participants, which might not show the statistical power necessary to yield a meaningful analysis and the interpretation of the results. Previously, differences between sexes contribute to variation in the obesity-related traits such as levels of fasting glucose and insulin were described. Lagou et al. (2021) indicated that fasting insulin in women shows stronger genetic correlations than in men with waist-to-hip ratio and anorexia nervosa [36]. Unfortunately, this study only included adult men, and thus we did not have the chance to compare the results between different age groups and genders. The participants were also relatively young, healthy, and physically active, which could have influenced the results, because systematic exercise reduces body weight [37]. Additionally, it should be emphasized that this is an observational study and that no causal mechanisms can be inferred.

## 5. Conclusions

The results of the present study suggest that the *FTO* gene, when tested individually, and all selected genes (*FTO*, *FABP2*, *LEP*, *LEPR*, and *MC4R*), when tested in gene–gene interactions, can modify body weight and composition parameters (BMI and FMI). We showed that harboring the *FTO* A allele might be associated with an over two times increased FMI. This observation constitutes the first important finding of the study, implying that harboring this specific allele is unfavorable for some individuals. The carriers of the A allele might have an increased accumulation of body fat and a higher risk of many obesity-related disorders. The results suggested that the *FTO* (rs9939609) polymorphism is a candidate marker for affecting body mass and body composition parameters in the Caucasian population. From the gene–gene interaction analysis, we established the second novel finding. Namely, all five studied polymorphisms are involved in the formation of obesity-related traits in the Caucasian population. The results imply that some individuals might benefit from carrying some combinations of genotypes (e.g., for BMI: AT × AA, TT × AA, TT × AG; for FMI: AT × GG, TT × AA, and TT × AG-*FTO* × *LEPR*, respectively), as regards the BMI and FMI. Additionally, the gene–gene interaction analysis confirmed that harboring the *FTO* T allele might be favorable for some individuals. This observation confirmed that the genetic risk of obesity is connected with the accumulation of numerous variants; thus, methods based on numerous SNPs are more informative than methods based on a single polymorphism. Understanding the genetics of obesity can extend our knowledge of diet individualization and exercise programs. It is crucial for the prevention of obesity-related diseases, supplementation and medical care.

## Figures and Tables

**Table 1:** Characteristics of the studied genes and polymorphic sites.

Gene	Chromosome Location	Gene Product	Variant	Functions of the Protein
*FTO*	16q12.2	2-oxoglutarate (2-OG) Fe (II) dependent demethylase	rs9939609; T > A	influencing the activity of pathways controlling daily food intake, nutrient preference, appetite, and satiety as well as control overeating
*FABP2*	4q26	fatty acid binding protein 2	rs1799883; G > A; Ala54Thr	participating in the absorption, intracellular transport, and metabolism of dietary fatty acids and their acyl-CoA esters in the small intestine
*LEP*	7q32.1	leptin	rs2167270; G > A;	regulating appetite by its inhibitory effects on food intake and increases in energy expenditure by stimulating the metabolism and physical activity
*LEPR*	1p31.3	leptin receptor	rs1137101; A > G; Gln223Arg	mediating leptin signaling
*MC4R*	18q21.32	melanocortin-4 receptor	rs17782313; T > C	the major regulator of food intake and energy expenditure

**Table 2:** Anthropometry and body composition of the participants.

Group	CON_BMI_ (*n* = 77)	OVER_BMI_ (*n* = 88)	*p*-Value	CON_FMI_ (*n* = 124)	OVER_FMI_ (*n* = 41)	*p*-Value
BMI (kg/m^2^)	23.4 ± 1.3	28.7 ±3.5	<0.01	24.6 ± 1.9	31.0 ± 3.9	<0.01
Age (years)	29.6 ± 7.2	34.8 ± 7.7	<0.01	31.3 ± 7.3	35.5 ± 7.3	<0.01
Height (cm)	180.2 ± 7.4	180.4 ± 6.4	0.81	180.0 ± 6.8	181.2 ± 7.0	0.34
Weight (kg)	76.1 ± 7.6	93.4 ± 13.5	<0.01	79.9 ± 8.4	101.8 ± 14.9	<0.01
FMI (kg/m^2^)	3.41 ± 1.0	6.5 ± 2.4	<0.01	4.0 ± 1.1	8.4 ± 2.2	<0.01
VTI (level)	3.2 ± 1.8	8.2 ± 3.6	<0.01	4.2 ± 2.2	10.9 ± 3.5	<0.01
Fat (%)	14.2 ± 3.7	22.1 ± 5.1	<0.01	15.8 ± 3.8	26.5 ± 3.7	<0.01

**Table 3:** SNP genotyping assays used in the study.

Gene	Variant	Assay ID
*FTO*	rs9939609	C__30090620_10
*FABP2*	rs1799883	C____761961_10
*LEP*	rs2167270	C__15966471_20
*LEPR*	rs1137101	C___8722581_10
*MC4R*	rs17782313	C__32667060_10

**Table 4:** The probability that the genotype frequencies do not differ from Hardy–Weinberg expectations and minor allele frequencies (MAF).

SNP	MAF(%)	ALL	OVER_BMI_	CON_BMI_	OVER_FMI_	CON_FMI_
*FTO* (rs9939609)	allele A (38.48)	0.87	0.82	1.00	1.00	1.00
*FABP2* (rs1799883)	allele T (23.94)	1.00	1.00	1.00	1.00	0.79
*LEP* (rs2167270)	allele A (46.36)	0.53	0.83	0.50	1.00	0.47
*LEPR* (rs1137101)	allele G (45.45)	0.88	0.83	1.00	1.00	0.86
*MC4R* (rs17782313)	allele C (20.00)	0.33	0.10	0.72	0.56	0.59

**Table 5:** Association analysis of the *FTO* (rs9939609) polymorphism with BMI.

Genetic Model	Genotype	OVER_BMI_	%	CON_BMI_	%	OR	95%CI		*p*-Value/*p*-Value */q-Value	AIC
Codominant	TT	32	36.4	31	40.3	1.00			0.74/0.84/0.62	233.4
	AT	41	46.6	36	46.8	1.10	0.57	2.15		
	AA	15	17.0	10	13.0	1.45	0.57	3.72		
Dominant	TT	32	36.4	31	40.3	1.00			0.61/0.56/0.58	231.7
	AT-AA	56	63.6	46	59.7	1.18	0.63	2.21		
Recessive	TT-AT	73	83.0	67	87.0	1.00			0.47/0.77/0.51	231.5
	AA	15	17.0	10	13.0	1.38	0.58	3.27		
Overdominant	TT-AA	47	53.4	41	53.2	1.00			0.98/0.72/0.69	232.0
	AT	41	46.6	36	46.8	0.99	0.54	1.83		
Alleles	T	105	59.66	98	63.64				0.53/-/0.54	
	A	71	40.34	56	36.36	1.82	0.84	1.89		

OR—odds ratio; 95% CI—confidence interval; AIC—Akaike information criterion; *p*-value *—adjusted by age *p*-value; q-value—FDR adjusted *p*-values.

**Table 6:** Association analysis of the *FTO* (rs9939609) polymorphism with FMI.

Genetic Model	Genotype	OVER_FMI_	%	CON_FMI_	%	OR	95%CI		*p*-Value/*p*-Value */q-Value	AIC
Codominant	TT	9	22.0	54	43.5	1.00			0.01/0.01/0.02	182.2
	AT	21	51.2	56	45.2	2.25	0.95	5.35		
	AA	11	26.8	14	11.3	4.71	1.63	13.59		
Dominant	TT	9	22.0	54	43.5	1.00			0.01/0.01/0.02	182.6
	AT-AA	32	78.0	70	56.5	2.74	1.21	6.23		
Recessive	TT-AT	30	73.2	110	88.7	1.00			0.02/0.04/0.04	183.8
	AA	11	26.8	14	11.3	2.88	1.19	6.99		
Overdominant	TT-AA	20	48.8	68	54.8	1.00			0.50/0.32/0.53	188.6
	AT	21	51.2	56	45.2	1.28	0.63	2.59		
Alleles	T	39	47.6	164	66.1				<0.01/-/0.02	
	A	43	52.4	84	33.9	2.15	1.26	3.69		

OR—odds ratio; 95% CI—confidence interval; AIC—Akaike information criterion; *p*-value *—adjusted by age *p*-value; q-value—FDR adjusted *p*-values.

**Table 7:** Best gene–gene interaction models, as identified by MDR.

Number of Loci	Best Combination	Division	Cross-Validation Consistency	Testing Balance Accuracy	*p*-Value
2	*FTO* × *LEPR*	BMI	4/10	0.59	0.02
	*FTO* × *LEPR*	FMI	6/10	0.63	<0.01
3	*LEP* × *LEPR* × *MC4R*	BMI	5/10	0.64	<0.01
	*FTO* × *LEP* × *LEPR*	FMI	6/10	0.70	<0.01
4	*FABP2* × *LEP* × *LEPR* × *MC4R*	BMI	10/10	0.72	<0.01
	*FTO* × *FABP2* × *LEP* × *LEPR*	FMI	5/10	0.75	<0.01
5	*FTO* × *FABP2* × *LEP* × *LEPR* × *MC4R*	BMI	10/10	0.78	<0.01
	*FTO* × *FABP2* × *LEP* × *LEPR* × *MC4R*	FMI	10/10	0.81	<0.01

## Data Availability

The data presented in this study are available on request from the corresponding author. The data are not publicly available due to privacy/ethical restrictions.
